# Mental health self-medication in psychiatry residents: from providing to seeking mental health care

**DOI:** 10.3389/fpubh.2025.1568455

**Published:** 2025-07-16

**Authors:** Diana Guízar-Sánchez, María Yoldi-Negrete, Carlos-Alfonso Tovilla-Zárate, Tecelli Domínguez-Martínez, Rebeca Robles-García, Ana Fresán

**Affiliations:** ^1^Department of Physiology, Faculty of Medicine, National Autonomous University of Mexico, Mexico City, Mexico; ^2^Clinical Research Directorate, Ramón de la Fuente Muñiz’ National Institute of Psychiatry, Ministry of Health, Mexico City, Mexico; ^3^Multidisciplinary Academic Division of Comalcalco, Juárez Autonomous University of Tabasco, Comalcalco, Tabasco, Mexico; ^4^Global Mental Health Research Center, ‘Ramón de la Fuente Muñiz’ National Institute of Psychiatry, Ministry of Health, Mexico City, Mexico

**Keywords:** psychiatry residents, self-medication, healthcare, mental health, medical culture

## Abstract

**Objective:**

The purpose of the present study was to determine the prevalence of self-medication among psychiatric residents with self-reported mental disorders, and to study the factors associated with self-medication.

**Methods:**

A cross-sectional retrospective study was conducted with psychiatry residents who answered an online survey. Variables were compared between psychiatry residents who do and do not self-medicate to treat self-reported mental health problems. Those that differed in the bivariate analyses were included in a multivariate logistic regression model to identify those associated with self-medication.

**Results:**

A total of 136 of the 330 psychiatry residents assessed comprised the final sample, 41.2% of which reported self-medication. Depression and anxiety were the most frequent mental health problems reported. Being verbally attacked (OR = 4.4), being in the last years of residency (OR = 4.2), being attacked by senior colleagues (OR = 3.7) and higher perceived discrimination (OR = 1.1) increased the risk for self-medication. Conversely, psychotherapy reduced the likelihood of self-medication (OR = 0.2).

**Conclusion:**

Self-medication is a common practice among psychiatric residents with mental disorders in Mexico. It is a major concern in psychiatry residents as it can cause symptom aggravation due to subjective medication. Health and educational institutions must protect residents from the risks of this practice by addressing the associated factors.

## 1 Introduction

The World Health Organization defines self-medication as “the selection and use of medicines by individuals to treat self-recognized disorders or symptoms” (1) or by health professionals such as the self-prescription of drugs not prescribed by another doctor (2), in other words, a colleague functioning as the attending physician. Self-medication is a worldwide phenomenon, with prevalences ranging from 11.2 to 93.7% and higher prevalences being observed in developing countries (3). These prevalences also vary according to the target population as well as the condition the health professional is attempting to treat. Nevertheless, self-medication is increasing worldwide and, in many cases, can lead to serious health consequences (4). Some authors consider that this increase could be due to the high cost of medical consultations, increased drug availability and easier access to medication through Internet and smartphones (5), together with lack of access to health care services (6).

Self-medication appears to be widely tolerated and condoned in specific situations (such as emergencies and geographic isolation) (7) and for health conditions that people consider do not require medical assistance, such as the treatment of flu-like symptoms, with suitable medicine in the correct dose (3). Nevertheless, it has been generally discouraged, as even symptoms of seemingly common health conditions require accurate, professional knowledge of symptoms that may be present in several medical conditions and appropriate treatment (8).

All patients are entitled to reasonable access to timely, competent care and are expected to seek appropriate medical help when they experience health problems. Medical help-seeking behavior is also to be expected when physicians experience problems with their own health. Self-medication by physicians and medical students is fairly common, being reported by more than 50% (9). However, self-medication deprives physician patients of the objectivity crucial to a high-quality clinical process and the empathy of a consulting physician (7). There is also a risk of overlooking an essential medical examination, diagnosis, and medical follow-up (9). Factors that have been found to lead to a higher likelihood of self-diagnosis and self-medication include lack of time, fear of rejection, unwillingness to bother another physician, fear of potentially exposing their weaknesses, unwillingness to ask for help, denial and the feeling that physicians should be able to manage their health problems (10, 11). It has also been considered that the reluctance to seek help is greater if the problem is associated with mental health (7).

There is a high prevalence of mental health problems, particularly anxiety and depression, among physicians and medical residents (12). Mental health professionals often act as “gatekeepers” in mental illness treatment and prevention. However, mental health practice does not provide passive immunity to mental illness. In the context of psychiatry residents, self-medication can assume specific forms due to the demanding, often stressful nature of psychiatric training. The intrinsic and extrinsic pressures of residency—such as the continuous emphasis on managing others’ psychological distress, intense academic workloads, self-stigma, social apathy, and difficulty finding time for personal medical care—can be overwhelming. These challenges, including psychological distress, depression, anxiety, and burnout, may drive residents to self-medicate in an effort to manage their symptoms (13).

Given the vulnerability of psychiatry residents to mental health issues and the access they have to psychiatric medication, self-medication can have serious implications for both their personal well-being and professional competence. Although their academic training gives them an advantage in regard to the use of psychiatric medication, diagnosis and treatment are based on their own knowledge and perception, without the objectivity of a certified psychiatrist with the experience and knowledge to make a diagnosis, provide the most suitable treatment and perform a clinical follow-up.

Despite the risks associated with self-medication, there are few studies describing the risk factors for self-medication among young physicians, and even fewer of psychiatrists in training who are prescribers. This retrospective study therefore sought to determine the prevalence of self-medication among psychiatric residents with self-reported mental disorders, and to study the factors associated with self-medication. We hypothesized that psychiatry residents reporting self-medication would: (1) be single, (2) be residents in the third and fourth year of residency, (3) have more residency-related adversity, and (4) have fewer individual protective factors. We also hypothesized that these variables would be associated with self-medication in psychiatry residents who self-reported mental health problems. To our knowledge, this is the first report comparing these features among psychiatry residents related to self-medication to treat self-reported mental health problems.

## 2 Materials and methods

This is an analytical, cross-sectional, retrospective study in which psychiatry residents participated in an online survey. The variables of interest were drawn from a larger dataset of a research project examining the physical and mental health of psychiatrists and psychiatry residents in Mexico.

### 2.1 Population

The response rate of our study was 45.8% of the 720 psychiatry residents registered at the time of the study in Mexico. A total of 330 psychiatry residents were assessed for eligibility. Within this group, 196 (59.4%) reported having a mental health problem, of which 159 (81.1%) were receiving treatment, with 136 (85.5%) receiving pharmacological treatment. Among the final sample of 136 psychiatry residents, 41.2% (*n* = 56) indicated that their pharmacological treatment had been self-medicated.

### 2.2 Data collection

The survey was composed of four sections, each of which is described in detail below:

The first section comprised demographic characteristics (age, sex, and marital status) at the time the resident answered the survey.

In the second section, *mental health features*, residents were asked about the presence of any mental health problem during their residency (major depression, anxiety disorders, burnout, suicide ideation, eating disorders, sleeping disorders, and trauma-related problems) and whether they had received specialized treatment for these conditions (whether psychotherapeutic or pharmacological). Only those who reported mental health problems and had received pharmacological treatment were included in the present study. Those who reported having received pharmacological treatment were asked whether this treatment had been prescribed by a mental health specialist or whether they had self-medicated.

The third section, *residency-related activities,* included the current year of medical specialization (first to fourth) and maximum working hours per day (excluding being on call and only including their regular work schedule). *Residency-related adversities* included having attended patients with severe suicide ideation, whether any of their patients had committed suicide, being attacked by patients, patients’ relatives or senior colleagues (all of the above with a yes/no answer), residency related-stress (assessed on a 100-point visual analog scale represented by a 100-mm-long horizontal line, with word descriptors at each end ranging from “0-no stress at all” to “100-maximum perceived stress” (14), and perceived discrimination. The latter was assessed using the 13 items on King’s Stigma Scale, adapted for medical specialists (15, 16), with higher scores reflecting higher perceived discrimination) and an adequate internal consistency (Cronbach’s alpha 0.84) for its use (16).

*The fourth section, Individual protective factors,* included being under psychotherapeutic treatment for self-reported mental health problems (yes/no answer), social support and self-efficacy for coping with stress. Social support was assessed with the Social Support Questionnaire (SSQ-6) comprising six items designed to measure the number of sources of support and satisfaction (1 = very dissatisfied to 6 = very satisfied) with the support provided by these people (17, 18). The SSQ-6 has an adequate reliability (Cronbach’s alpha >0.84 for its use in Mexican population) (18). Self-efficacy was evaluated with the Perceived Self-Efficacy Scale for Coping with Stress (19), comprising eight items (1 = totally disagree to 5 = totally agree), assessing efficacy and outcome expectations was also used. The total score of the scale (obtained from the sum of these two dimensions) was used in the present study, with higher scores reflecting greater confidence in one’s ability to cope with stress. This scale has adequate reliability with a Cronbach’s alpha of 0.75 (19).

The survey took approximately 25 to 30 min to complete and was conducted in Spanish. The recruitment procedure was performed using a non-probabilistic convenience sample approach with psychiatry residents in Mexico. Residents were invited to participate through social media (Twitter and Facebook) and a link to the online survey at the end of the 2018 annual residency exam of the Faculty of Medicine of the National Autonomous University of Mexico (Universidad Nacional Autónoma de México), UNAM.

Residents were also informed that they could withdraw from the study by dropping out of the survey and guaranteed that the information collected until that point would be eliminated from the study. After this information had been provided, participants who voluntarily agreed to participate began completing the survey.

### 2.3 Ethical considerations

The aims and procedures of the study were approved by the Ethics and Research Committee (No. 09-CEI-010-20170316) of the Ramón de la Fuente Muñiz National Institute of Psychiatry of the Mexican Health Ministry in Mexico City and all participants provided informed consent. At the beginning of the survey, the researchers provided an explanation of the nature and procedures of the study, ensuring the anonymity of the data, which would only be used for research purposes.

### 2.4 Data analysis

Frequencies and percentages for categorical variables and means and standard deviations (SD) for continuous variables were used for the descriptive analysis. Chi-square tests (χ^2^) and independent sample *t*-tests were used for the comparison between residents who self-medicated to treat reported mental health problems (SM) and those who did not (NSM). A logistic regression model with the backward stepwise modeling approach was performed to determine which variables (demographic characteristics, residency-related activities and adversities, and individual protective factors) were associated with self-medication. Tolerance and variance inflation factor (VIF) values measuring collinearity were calculated for the regression model, with tolerance values ranging from 0.1 to 0.98 and VIF values from 1.0 to 2.4, which were within the acceptable ranges. We reported the first initial regression modeling with all the variables where differences were found in the bivariate analysis, and the final model with only the variables that remained significant after the backward stepwise process. The alpha value for all tests was set at *p* < 0.05 (for both groups). All analyses were performed with the IBM SPSS Statistics for Windows, Version 25.0. Armonk, NY, United States: IBM Corp.

## 3 Results

### 3.1 Demographic characteristics and mental health features

The sample comprised a majority of women (66.9%) and single residents (90.4%) with a mean age of 28.5 years (SD = 2.1), with no differences being found between residents who self-medicated (SM) and those who did not (NSM) (see Table 1).

**Table 1:** Demographics and mental health features among psychiatry residents who do and do not self-medicate to treat reported mental health problems.

	Total *n* = 136		NSM group^+^ *n* = 80		SM group* n = 56		Statistics
Demographic features							
Gender—n (%)							
Men	45	33.1	25	31.3	20	35.7	χ^2^ = 0.2, *p* = 0.58
Women	91	66.9	55	68.8	36	64.3	
Age ^a^	28.5	2.1	28.4	2.3	28.6	1.7	*t* = −0.6, *p* = 0.49
Marital status—n (%)							
Single	123	90.4	73	91.3	50	89.3	χ^2^ = 0.1, *p* = 0.70
Partnered	13	9.6	7	8.8	6	40.7	
Mental health features							
Depression—Yes	112	82.4	65	81.3	47	83.9	χ^2^ = 0.1, *p* = 0.68
Anxiety—Yes	111	81.6	68	85.0	43	76.8	χ^2^ = 1.4, *p* = 0.22
Burnout—Yes	67	49.3	39	48.8	28	50.0	χ^2^ = 0.02, *p* = 0.88
Suicide ideation—Yes	32	23.5	17	21.3	15	26.8	χ^2^ = 0.5, *p* = 0.45
Eating disorders—Yes	15	11.0	8	10.0	7	12.5	χ^2^ = 0.2, *p* = 0.64
Sleeping disorders—Yes	70	51.5	42	52.5	28	50.0	χ^2^ = 0.08, *p* = 0.77
Trauma-related disorders—Yes	11	8.1	4	5.0	7	12.5	χ^2^ = 2.4, *p* = 0.11

^+^Non-self-medicated group. *Self-medicated group. ^a^Data presented in means and SD.

The most frequent mental health problems reported by residents were depression (82.4%), anxiety (81.6%), sleeping disorders (51.5%), and burnout (49.3%). As can be seen in Table 1, the frequency of self-reported mental health problems was similar in the SM and NSM groups.

### 3.2 Residency-related activities and adversities

Fifty-four residents (39.7%) were in their third year of residency, followed by 42 (30.9%) in the second, 32 (23.5%) in the first, and eight (5.9%) in the fourth year. A higher proportion of residents from the last years of residency (third and fourth) reported having self-medicated to treat self-reported mental health problems (Table 2). Residents in both groups reported a similar number of working hours.

**Table 2:** Residency-related activities and adversities, and individual protective factors among psychiatry residents who do and do not self-medicate to treat self-reported mental-health problems.

	Total *n* = 136		NSM group^+^ *n* = 80		SM group* n = 56		Statistics
Residency-related activities							
Year of residency—n (%)							
First and second year	74	54.4	50	62.5	24	42.9	χ^2^ = 5.1, *p* = 0.02
Third and fourth year	62	45.6	30	37.5	32	57.1	
Hours worked per day ^a^	13.5	6.9	12.7	6.5	14.7	7.8	*t* = −1.6, *p* = 0.10
Residency-related adversities							
Patients with suicide ideation—Yes	122	89.7	72	90.0	50	89.3	χ^2^ = 0.01, *p* = 0.89
Patients who committed suicide—Yes	36	26.5	23	28.8	13	23.1	χ^2^ = 0.5, *p* = 0.47
Verbally attacked—Yes	52	38.2	25	31.3	27	48.2	χ^2^ = 4.0, *p* = 0.04
Physically attacked—Yes	40	29.4	17	21.3	23	41.1	χ^2^ = 6.2, *p* = 0.01
Psychologically attacked—Yes	39	28.7	19	23.8	20	35.7	χ^2^ = 2.3, *p* = 0.12
Attacked by patients—Yes	54	39.7	28	35.0	26	46.4	χ^2^ = 1.7, *p* = 0.18
Attacked by patients’ relatives—Yes	44	32.4	19	23.8	25	44.6	χ^2^ = 6.5, *p* = 0.01
Attacked by senior colleagues—Yes	40	29.4	16	20.0	24	42.9	χ^2^ = 8.2, *p* = 0.004
Residency-related stress ^a^	60.8	24.6	61.1	23.5	60.2	26.3	*t* = 0.2, *p* = 0.83
Perceived discrimination ^a^	33.2	11.8	31.3	11.5	36.0	11.7	*t* = −2.3, *p* = 0.02
Individual protective factors							
Psychotherapy—Yes	96	70.6	63	78.8	33	58.9	χ^2^ = 6.2, *p* = 0.01
Sources of support (number) ^a^	2.9	1.7	3.2	1.8	2.6	1.5	*t* = 1.9, *p* = 0.06
Satisfaction with social support ^a^	4.7	1.5	4.8	1.6	4.6	1.3	*t* = 0.5, *p* = 0.61
Self-efficacy for coping with stress ^a^	24.3	3.4	24.4	2.9	24.2	4.0	*t* = 0.3, *p* = 0.75

^+^Non self-medicated group. *Self-medicated group. ^a^Data presented in means and SD.

Regarding residency-related adversities, both groups reported a similar percentage of care of patients with suicide ideation and those who committed suicide. Also, residency-related stress was similar in both groups (with mean scores of 61.1 and 60.2). Nevertheless, residents in the SM group reported physical and verbal attacks more frequently than residents in the NSM. Patients’ relatives and senior colleagues were more frequently identified as aggressors by residents in the SM group and perceived discrimination was higher in this group (see Table 2).

### 3.3 Individual protective factors

In addition to pharmacological treatment, 70.6% (*n* = 96) of residents were under psychotherapy, with more residents in the NSM group receiving pharmacological and psychotherapeutic treatment. Similar scores were reported in both groups regarding the number of sources of social support, satisfaction with current social support and perceived self-efficacy for coping with stress (Table 2).

### 3.4 Variables associated with self-medication in psychiatry residents

The comparative analysis showed that residents in the SM group were mostly in the last years of the psychiatry residency, more likely to report physical and verbal aggression by patients’ relatives and senior colleagues, had higher perceived discrimination and received psychotherapy for their self-reported mental health problems less frequently.

The most important variables associated with self-medication were: (1) being verbally attacked (OR = 4.4), (2), being in the last years of the residency (OR = 4.2), (3) being attacked by senior colleagues (OR = 3.7) and higher perceived-discrimination scores (OR = 1.1). Being under psychotherapeutic treatment reduces the probabilities of self-medication (OR = 0.2). This model explains 37.1% of the variance and is adequate according to the goodness-of-fit-test (*p* = 0.8) (see Table 3).

**Table 3:** Factors associated with self-medication in psychiatry residents.

	Initial model			Final model		
	OR	95% CI		OR	95% CI	
Residency-related activities and adversities						
Year of residency—3rd and 4th	4.1**	1.7	10.1	4.2**	1.7	10.2
Verbally attacked—Yes	3.0	0.6	14.4	4.4*	1.2	15.1
Physically attacked—Yes	1.6	0.4	5.9	–	–	–
Attacked by patients’ relatives	1.1	0.33	4.1	–	–	–
Attacked by senior colleagues—Yes	3.1	0.8	11.4	3.7*	1.2	11.3
Perceived discrimination	1.1***	1.04	1.1	1.1***	1.04	1.1
Individual protective factors						
Psychotherapy—Yes	0.2***	0.07	0.53	0.2***	0.07	0.53

R^2^ Nagelkerke = 0.37, R^2^ Nagelkerke = 0.37. * *p* ≤ 0.05, **p ≤ 0.01, ***p ≤ 0.001 Hyphen (−) indicates variables that did not enter the regression model.

## 4 Discussion

The response rate of our study was high in accordance to the total psychiatry residents registered at the time of the study in Mexico (20). It should be noted that over half the residents reported having had a mental health problem during their residency, which is consistent with previous studies (21, 22). Data from a national survey in France found that psychiatry residents are more vulnerable to depression and anxiety (23), which is in line with the high percentages of depression and anxiety reported by the residents in the present study.

In our study, near half of psychiatry residents on pharmacological treatment had self-medicated, midway between what has been reported by other studies of residents in various specialties (24) (30% in all residents and 26% in psychiatry residents) and among Swedish physicians (60%) (25). Psychiatry residents use more antidepressants and anxiolytics to treat the symptoms affecting them than other specialties (22). Although we do not know the specific medication used by the residents in the present study, antidepressants and anxiolytics were probably the most commonly used ones due to the high prevalence of depression and anxiety reported.

A significant association was found between self-medication and being in the last years of the specialty training. The World Medical Association states that responsible self-medication requires the use of appropriate doses of non-prescription medications with proven safety, quality, efficacy, and use for an indicated condition (26). This definition implies knowledge of the use of medication. Residents in the last years of their psychiatry residency have greater knowledge and understanding of psychiatric medication, which may make them more confident about self-diagnosis and its pharmacological management (27). Nevertheless, self-reported mental health problems may not meet clearly defined diagnostic criteria or an objective symptoms assessment that leads to responsible self-medication in psychiatry residents. Moreover, qualitative studies have shown that self-diagnosis of mental health conditions is biased in physicians as they tend to under-or over-react to their symptoms, fluctuating from diagnosing disorders with the worst prognosis to ruling out a disorder altogether (28).

Verbal aggression, particularly from senior colleagues, also leads to self-medication in psychiatry residents. This is more frequently observed in medical students than in those from other faculties and supposedly justified by the fact that they have a high degree of responsibility for the health and lives of other people (12). Aggression from peers or higher-ranking colleagues has been associated with negative mental health outcomes and a negative teaching environment which may prevent residents from raising questions and concerns during their residency, making them feel insecure and impeding the learning process (29). The higher perceived discrimination and stigma among residents who self-medicate is likely to contribute to the self-medication of psychiatry residents with mental health problems. Residents may prefer to self-medicate than be exposed to discrimination, lack of compassion or be deemed incompetent by colleagues or even feel their residency is at risk due to these symptoms (13, 30). Conversely, a supportive teaching environment could potentially counter the stigma associated with professional care seeking, and therefore potentially lower the frequency of self-medication associated with mental health issues (25, 31).

The importance of psychotherapy in preventing self-medication cannot be overstated. Self-medication implies avoiding seeking help due to mental health issues, while receiving psychotherapy is clearly related to adequate mental health-seeking behavior with a trained mental health provider. In general, people with mental health problems prefer psychological treatment to medication (32), which is also regarded as protection against stress, burnout and anxiety symptoms as a result of residency training (33, 34). However, this may not be the case for psychiatry residents. Although personal psychotherapy is mandatory during psychiatry residency in several countries (35), this is not the case in Mexico. In addition, psychiatry residents face several challenges to receive this treatment, particularly finding the time to attend psychotherapy sessions without interfering with their residency activities, and the cost. Many residents view self-medication as the solution, even though psychotherapy with a mental health professional who objectively evaluated the symptoms and distress each resident presents and provided the support and treatment they required (33) would obviate the need for the use of psychiatric medications.

The present study has limitations that must be mentioned. Although the sample size of our study is adequate, our results cannot be generalized to all psychiatry residents in Mexico. Residents with a specific interest in their mental health may have been more motivated to answer the survey while those who were more distressed or impaired by mental health problems may have declined to participate in the study. Moreover, the survey did not include the psychiatric medication used by residents or the theoretical approach of the psychotherapy they received, both which should be addressed in future studies. The cross-sectional design of the study prevents us from determining whether pharmacological treatment, whether self-prescribed or not, as well as psychotherapy, were helpful for the mental health problems experienced by the residents. Also, the retrospective design of the study does not allow us to determine a causal link and therefore, our results should be interpreted as associated factors to self-medication. Longitudinal studies should be performed to determine the impact of self-medication on the mental health of psychiatry residents. While residents were asked to indicate whether the mental health condition was present during their residency, no precise information on the onset—such as date or age—was obtained, leaving open the possibility that the condition began before residency. Finally, although self-diagnosis entails a significant bias, psychiatry residents have the knowledge of symptoms and clinical course of psychiatric disorders. The percentages of self-reported mental health problems and self-medication are therefore a cause for concern.

Our study acknowledges the importance of the recognition of self-medication for mental health problems in psychiatry residents. Even though this population has knowledge of psychiatric medications, self-medication is a pressing concern as psychiatry residents are still in academic training and unable to be objective in assessing their own symptoms. This can cause potential symptom aggravation or lack of improvement due to inappropriate, subjective medication (25).

## 5 Conclusion

The medical culture must realize that psychiatry residents may be affected by the challenges they face during their training. Health and educational institutions must protect residents from the risks posed by self-medication. This issue must be included in medical curricula as well as the mentoring residents receive at the health institutions where they complete their residencies.

Since psychiatry residents are future psychiatrists who will be taking care of the mental health of the population, their mental health is important. It is therefore essential to foster self-care, integrity, self-reflection, and the ability to admit weaknesses and mistakes in psychiatry residents while striving for continuous improvement and learning.

## Data Availability

The raw data supporting the conclusions of this article will be made available by the authors, without undue reservation.
